# Geriatric nutritional risk index as a predictor of mortality in women with chronic inflammatory airway disease: evidence from NHANES 1999–2018

**DOI:** 10.3389/fnut.2025.1547952

**Published:** 2025-03-10

**Authors:** Zhao Chen, YouLi Wen, Wenqiang Li, Jingshan Bai, Peng Zhou, Qian He, Zhiping Deng

**Affiliations:** ^1^Department of Respiratory and Critical Care Medicine, Zigong First People's Hospital, Zigong, China; ^2^Department of Respiratory and Critical Care Medicine, Xiong’an Xuanwu Hospital, Xiongan New Area, China; ^3^Department of Basic Medical Sciences, Changsha Medical University, Changsha, China; ^4^Key Laboratory of Birth Defects and Related Diseases of Women and Children (Sichuan University) of Ministry of Education, Department of Obstetrics and Gynecology, West China Second University Hospital, Sichuan University, Chengdu, China

**Keywords:** cardiovascular disease, CIAD, cox regression analysis, GNRI, NHANES

## Abstract

**Background:**

The incidence of Chronic Inflammatory Airway Diseases (CIAD) has been steadily increasing, making it a significant contributor to the global disease burden. Additionally, the risk of airway diseases in elderly women continues to rise each year, with nutritional factors playing a crucial role in the progression of CIAD. The Geriatric Nutritional Risk Index (GNRI) is a novel tool for assessing individual nutritional status. This study aims to assess the relationship between GNRI and the risk of all-cause and cardiovascular mortality in elderly women with CIAD, providing guidance for nutritional interventions to reduce mortality risk.

**Methods:**

Data from elderly female patients and relevant indicators were sourced from the National Health and Nutrition Examination Survey (NHANES) database. Nutritional status was assessed using the GNRI, and patients were divided into four groups based on their GNRI quartiles. Weighted Cox proportional hazards regression models were used to examine the relationship between GNRI and all-cause as well as cardiovascular mortality in elderly women with CIAD. Additionally, restricted cubic spline (RCS) analysis was applied to explore the association between GNRI and different mortality outcomes, and subgroup analysis was conducted to further validate the robustness of the findings.

**Results:**

A total of 1,417 elderly female CIAD patients were included in this study. During a median follow-up of 91 months, 515 deaths from all causes and 157 deaths from cardiovascular causes occurred. Multivariable-adjusted Cox proportional hazards models indicated that compared to the lowest GNRI quartile, the other quartiles showed a general decreasing trend in both all-cause and cardiovascular mortality risk (*p* < 0.05). In the fully adjusted model, the highest GNRI quartile had the lowest risks of all-cause mortality (HR = 0.40, 95% CI: 0.22–0.72, *p* < 0.05) and cardiovascular mortality (HR = 0.29, 95% CI: 0.11–0.78, *p* < 0.05).The RCS analysis demonstrated a nonlinear association between GNRI and both all-cause and cardiovascular mortality (*P* for nonlinearity <0.001).

**Conclusion:**

In elderly women with CIAD, lower GNRI levels are associated with an increased mortality risk. GNRI may serve as a potential predictive tool for both all-cause and cardiovascular mortality, providing valuable insights for nutritional interventions and clinical decision-making.

## 1 Introduction

Chronic inflammatory airway diseases (CIAD), such as asthma, chronic bronchitis, and chronic obstructive pulmonary disease (COPD), have emerged as a significant global public health problem due to their increasing role in the worldwide disease burden (1). Key risk factors for CIAD include tobacco exposure, air pollution, and environmental particulate matter (1). In 2017, chronic respiratory diseases were responsible for approximately 3.91 million deaths globally, accounting for 7.0% of total mortality and representing an 18% increase from 1990 (2). COPD and asthma are the most prevalent CIADs, both associated with chronic airway inflammation (3, 4). Asthma, affecting over 300 million people worldwide, led to 455,000 deaths, while COPD, projected to impact 212.3 million people, caused 3.3 million deaths (5, 6). Gender disparities in the burden of CIAD are well-established, with studies showing higher rates of COPD among women in both Canada and the U.S. (7–9). As the prevalence of CIAD increases among elderly women, it is essential to develop more effective interventions tailored to this demographic to reduce the growing disease burden and associated healthcare costs.

Malnutrition significantly influences the development and progression of Chronic Inflammatory Airway Diseases (CIAD) in the elderly, with recent studies highlighting its critical role. Malnutrition is prevalent among hospitalized elderly patients, affecting approximately 30–60% of those with Chronic Obstructive Pulmonary Disease (COPD) (10). This condition adversely impacts various health outcomes, including diminished exercise tolerance, increased hospitalization rates, airway obstruction, and elevated mortality risk (10). Incorporating nutritional status assessments into the screening and management of CIAD in the elderly is essential. The immune system’s regulation of nutritional status involves mechanisms such as inflammation and oxidative stress. Proinflammatory cytokines can interact with glucagon-like peptide-1 (GLP-1) (11), leading to weight loss, while albumin levels often serve as indicators of nutritional status.

Recent research underscores the association between malnutrition and adverse outcomes in elderly patients with CIAD. A study published in Scientific Reports found that malnutrition is a risk factor negatively affecting clinical outcomes, including increased mortality rates, in elderly patients (12). Additionally, a study in Clinical Nutrition reported that malnutrition in older adults is linked to higher all-cause mortality rates, emphasizing the need for early identification and intervention (13).

Despite these findings, few studies have examined the connection between overall nutritional status and the prognosis of chronic inflammatory airway disorders. The complex interplay between malnutrition, inflammation, and oxidative stress in the elderly necessitates further research to develop effective nutritional interventions aimed at improving health outcomes in this population.

The Geriatric Nutritional Risk Index (GNRI) is a clinically accessible metric that uses readily available data to objectively calculate malnutrition risk (14). Derived from serum albumin levels and BMI, it can predict complications resulting from malnutrition (15). Research has demonstrated a strong correlation between GNRI and the prognosis of various illnesses, such as diabetes, heart failure, cancer, and osteoporosis (16). Several studies have shown that a lower GNRI is associated with a higher risk of death (17–19). However, it remains unclear whether GNRI is a reliable indicator of malnutrition in individuals with chronic inflammatory airway disorders and how it impacts prognosis.

This study examines the relationship between the GNRI and mortality risk in women aged 60 years and older with CIAD, utilizing data from the National Health and Nutrition Examination Survey (NHANES). By evaluating the prognostic significance of nutritional status in predicting disease-specific mortality, the research aims to inform early nutritional intervention strategies that could potentially improve clinical outcomes within this vulnerable population.

## 2 Methods

### 2.1 Study methods and population

This study utilized data from the National Health and Nutrition Examination Survey (NHANES), a large cross-sectional study designed to assess the nutritional status of non-institutionalized Americans. Data was collected through laboratory tests conducted at mobile centers, physical examinations, and structured home interviews. The study employed a multistage probability sampling method to select a representative sample of the American population, with surveys conducted every 2 years and follow-ups every 4 years. Detailed procedures are available on the official website (http://www.cdc.gov/nchs/nhanes.htm, accessed October 1, 2023). The protocols for NHANES have been approved by the Research Ethics Review Board of the National Center for Health Statistics (NCHS), and written informed consent was obtained from all participants in the study.^1^

**Figure 1:**
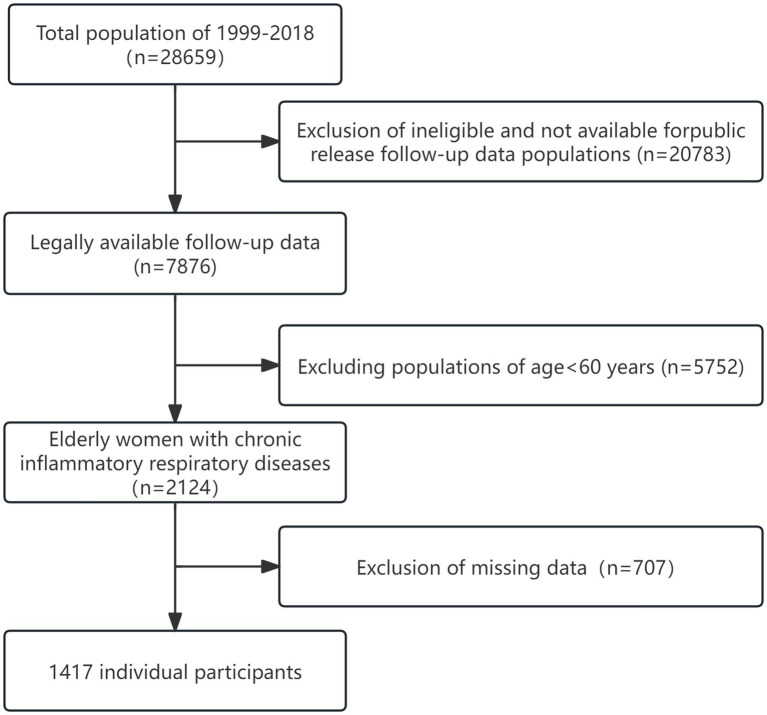
Study flowchart outlining participant selection and exclusion criteria.

For this study, we utilized NHANES data from 1999 to 2018. Patients were excluded if they were lost to follow-up, had incomplete data, were under 60 years of age, or had missing covariates. After applying the inclusion and exclusion criteria, the final analysis included 1,417 CIAD patients aged 60 years or older. A flowchart illustrating the inclusion and exclusion process is shown in Figure 1.

### 2.2 Definition of GNRI and CIAD

The subject’s height (in meters), weight (in kilograms), ideal weight (in kilograms), and blood albumin level (in grams per liter) are used to calculate the GNRI (14, 20).The GNRI is calculated using the following formula:


$$\begin{array}{l} \mathrm{GNRI}=1.489\times \mathrm{albumin}\;\left(g/L\right)+41.7\times \\ \left(\mathrm{ideal weight}/\mathrm{current weight}\right)\text{.} \end{array}$$

where the ideal weight is calculated as 22 × height (m)2. If the current weight exceeds the ideal weight, the weight-to-ideal weight ratio is set to 1 (14, 21).

CIAD in this study include COPD, asthma, and chronic bronchitis. Data regarding these conditions were obtained from the NHANES questionnaire, which asked participants whether they had ever been diagnosed with COPD, asthma, or chronic bronchitis by a physician or other healthcare provider. CIAD was identified in participants who answered “yes” to any of these questions.

### 2.3 Mortality risk in the study population

The primary endpoints of this study were all-cause mortality and cardiovascular mortality, determined using mortality risk-related records that documented any cause of death. Mortality risk information was obtained from the National Death Index (NDI) database.^2^ According to the most recent update from the NDI database, each participant’s follow-up period extended from the date of enrollment until their death or December 31, 2019, whichever occurred first. Cardiovascular mortality cases were identified using the International Statistical Classification of Diseases, 10th Revision (ICD-10) codes (I00-I09, I11, I13, and I20-I51).

### 2.4 Covariates

The study’s variables were drawn from the NHANES survey’s demographic and health questionnaires and were divided into three primary categories: laboratory test results, physical examination measures, and demographic traits. Demographic characteristics were assessed using standardized surveys and included age, race, marital status, education level, family income-to-poverty ratio (PIR), health insurance coverage, smoking status, and alcohol consumption. Physical examination parameters, including Body Mass Index (BMI), were obtained at the Mobile Examination Centers (MECs). Participants were instructed to fast for at least 8.5 h but no more than 24 h in order to obtain laboratory data. Blood tests included neutrophil counts and serum creatinine levels.

Age was categorized as 60–70 years and ≥ 70 years; race was classified as White or other; marital status was categorized as married/spouse, divorced/widowed/separated, and unmarried; education levels were classified as less than high school, high school or equivalent, and college or above. PIR was defined as the ratio of family income to the poverty line, categorized into lower income (<1), middle income (1 ≤ PIR ≤ 3), and higher income (>3) (22, 23).

Based on survey replies on whether or not participants had obtained family insurance, health insurance status was ascertained. There were three categories for smoking status: never smoked (less than 100 cigarettes), former smoker (more than 100 cigarettes but no longer smoking), and current smoker (more than 100 cigarettes and smoking on certain days or every day) (24). Never drank (defined as consuming less than 12 drinks in a lifetime) and drank (defined as consuming 12 or more drinks in a lifetime) were the two categories for alcohol consumption (25).

Weight (kg) divided by height (m) squared was used to determine BMI, which was then used to classify people as normal (<25 kg/m2), overweight (25 ≤ BMI < 30 kg/m2), and obese (≥30 kg/m2) (26). Diabetes was identified if participants met any of the following criteria: prior diagnosis, HbA1c level ≥ 6.5%, fasting glucose ≥7.0 mmol/L, random plasma glucose ≥11.1 mmol/L, 2-h OGTT ≥11.1 mmol/L, or use of antidiabetic medication. When standard reference levels were exceeded yet the aforementioned requirements were not met, impaired glucose control was identified.

Participants were asked if they had been diagnosed with congestive heart failure, coronary artery disease, angina, myocardial infarction, or stroke by a physician or other healthcare provider in order to ascertain their cardiovascular disease (CVD) status. Self-reported hypertension (HTN) or the use of antihypertensive drugs were considered forms of HTN. The American Heart Association/American College of Cardiology (AHA/ACC) 2017 guidelines for the monitoring and diagnosis of hypertension were used to determine HTN for those who did not self-report. Participants were considered hypertensive if their systolic blood pressure was ≥130 mmHg or their diastolic blood pressure was ≥80 mmHg (27). Tumor status was assessed through self-reporting by the participants.

### 2.5 Statistical analysis

The computations used sample weights, stratification, and primary sampling units to adjust for non-responses, oversampling of specific subgroups, and unequal sampling probabilities in compliance with the NHANES analytical standards. The sampling weights were calculated using the following formulas: to calculate the 2-year fasting subsample weights for 2003–2018, multiply the 2-year fasting subsample weights by 1/10, and to calculate the 10-year fasting subsample weights for 1999–2002, multiply the 4-year fasting subsample weights by 2/10.

R (version 4.3.1) was the statistical program used for all analyses. The “nhanesR” program was used for data extraction and analysis. Four quartiles were identified from the GNRI: Q1 (80–110), Q2 (110–117.6), Q3 (117.6–127.6), and Q4 (127.6–212.4). Continuous variables were given as weighted means (standard errors), and weighted t-tests were used to compare baseline features, such as means and proportions. Additionally, categorical variables—which were represented as weighted frequencies and percentages—were compared using the Rao-Scott chi-square test.

It was determined that the Q1 group (GNRI 80–110) was typical. Cox proportional hazards regression models were used to examine the relationship between GNRI and the risk of cardiovascular and all-cause death in both crude and adjusted models. The results were presented as hazard ratios (HRs) and 95% confidence intervals (CIs). Using restricted cubic spline (RCS) curves, the association between GNRI and cardiovascular and all-cause mortality was further evaluated after controlling for a number of factors. Additionally, subgroup analyses were carried out according to age, race, smoking status, education level, and alcohol use. A two-sided *p*-value<0.05 was considered statistically significant.

## 3 Results

### 3.1 Characteristics of the study population

Table 1 presents the basic characteristics of the study population. This study included 1,417 elderly female patients with CIAD, with a median follow-up duration of 91 months. Significant differences in age, smoking status, BMI, diabetes, hypertension, and neutrophil count were observed among the various subgroups (*p* < 0.05).

**Table 1:** Basic characteristics.

Characteristic	Overall	Q1	Q2	Q3	Q4	*p value*
	*n* = 1,417 (%)	*n* = 354 (%)	*n* = 354 (%)	*n* = 354 (%)	*n* = 355 (%)	
Age group						< 0.0001
60-70 years	736(54.38)	142(45.16)	163(46.26)	199(58.30)	232(68.15)	
≥70 years	681(45.62)	212(54.84)	191(53.74)	155(41.70)	123(31.85)	
Race/ethnicity						0.32
White race	817(82.88)	235(85.66)	214(83.01)	184(81.39)	184(81.27)	
Other race	600(17.12)	119(14.34)	140(16.99)	170(18.61)	171(18.73)	
Marital status						0.28
Married/with spouse	620(49.88)	144(46.11)	155(51.44)	172(54.78)	149(47.66)	
Divorced/widowed/living alone	746(47.16)	199(51.93)	189(45.83)	170(42.36)	188(48.04)	
Never married	51(2.96)	11(1.97)	10(2.73)	12(2.86)	18(4.30)	
Educational attainment						0.71
Junior high school or below	455(22.41)	116(24.02)	104(22.70)	103(19.01)	132(23.65)	
High school or technical secondary school	363(27.99)	84(24.75)	96(30.15)	100(29.16)	83(28.13)	
College or above	599(49.60)	154(51.23)	154(47.15)	151(51.83)	140(48.21)	
Poverty income ratio group						0.09
1	483(24.02)	107(22.83)	114(20.18)	117(22.94)	145(30.00)	
2	594(42.22)	155(41.12)	161(48.55)	142(38.85)	136(40.31)	
3	340(33.76)	92(36.04)	79(31.27)	95(38.21)	74(29.69)	
Health insurance						0.97
Insured	1,332(95.40)	336(95.30)	333(95.38)	331(95.94)	332(95.04)	
Uninsured	85(4.60)	18(4.70)	21(4.62)	23(4.06)	23(4.96)	
Smoking status						< 0.001
Non-smoker	693(46.41)	152(41.21)	180(50.58)	192(47.18)	169(46.95)	
Former smoker	518(38.29)	122(34.58)	122(33.23)	128(42.19)	146(43.42)	
Current smoker	206(15.30)	80(24.21)	52(16.19)	34(10.63)	40(9.63)	
Alcohol consumption status						0.2
Yes	1,109(82.86)	291(86.24)	277(80.78)	263(80.18)	278(83.91)	
No	308(17.14)	63(13.76)	77(19.22)	91(19.82)	77(16.09)	
BMI(kg/m^2^)						< 0.0001
Normal (< 25)	331(24.78)	262(76.90)	67(18.62)	2(0.39)	0(0.00)	
Overweight (25–30)	431(30.36)	88(22.30)	232(66.77)	111(33.50)	0(0.00)	
Obese (≥ 30)	655(44.86)	4(0.79)	55(14.61)	241(66.12)	355(100.00)	
Diabetes						0.001
Yes	341(19.24)	45(8.80)	75(18.22)	96(21.95)	125(28.46)	
Boardline	62(4.02)	12(2.53)	17(4.52)	14(4.22)	19(4.88)	
No	1,014(76.74)	297(88.68)	262(77.26)	244(73.83)	211(66.66)	
Hypertension						< 0.0001
Yes	967(64.50)	211(54.36)	231(64.58)	244(64.70)	281(74.66)	
No	450(35.50)	143(45.64)	123(35.42)	110(35.30)	74(25.34)	
Cancer						0.08
No	1,108(72.85)	256(66.65)	281(73.22)	280(73.54)	291(78.23)	
Yes	309(27.15)	98(33.35)	73(26.78)	74(26.46)	64(21.77)	
CVD						0.78
Yes	407(27.42)	103(26.01)	97(25.97)	94(28.24)	113(29.54)	
No	1,010(72.58)	251(73.99)	257(74.03)	260(71.76)	242(70.46)	
Laboratory						
Scr (umol/L)	74.81(61.39–87.92)	74.94(61.39–86.32)	76.19(62.31–88.16)	74.81(61.16–87.67)	73.72(57.96–89.17)	0.65
Neu (10^9^L)	4.20(3.30–5.30)	4.10(3.20–5.10)	3.90(3.20–4.90)	4.20(3.30–5.20)	4.60(3.60–5.70)	< 0.001

### 3.2 Relationship between GNRI and the risk of all-cause and cardiovascular mortality

Among the 1,417 senior female patients with CIAD, 515 died from all causes, and 157 from cardiovascular causes, during a median follow-up period of 91 months. To investigate the relationship between GNRI and mortality risk, three multivariable Cox regression models were developed. In Model 1, only age and race were adjusted. Model 2 included the covariates from Model 1, with additional adjustments for the poverty-to-income ratio (PIR), insurance status, smoking, education level, and alcohol consumption. Model 3 further adjusted for body mass index (BMI), cardiovascular disease, hypertension, diabetes, serum creatinine levels, and neutrophil count, based on the covariates from Model 2.

The results showed that higher GNRI levels were generally associated with a lower risk of both all-cause and cardiovascular mortality across all three models. In the fully adjusted model, compared to the lowest GNRI quartile, the odds ratios (ORs) and 95% confidence intervals (CIs) for all-cause mortality in the higher GNRI quartiles were 0.60 (0.44–0.84), 0.54 (0.32–0.90), and 0.40 (0.22–0.72) (*p* < 0.05), respectively. Similarly, for cardiovascular mortality, the ORs and 95% CIs were 0.59 (0.37–0.93), 0.33 (0.12–0.85), and 0.29 (0.11–0.78) (*p* < 0.05), respectively. These findings suggest that, after adjusting for multiple confounders, higher GNRI values are associated with a reduced risk of both all-cause and cardiovascular mortality (Table 2).

**Table 2:** GNRI and risk of mortality in elderly women with CIAD.

Characteristic	Model 1	*P for trend*	Model 2	*P for trend*	Model 3	*P for trend*
	*HR* (95%*CI*)		*HR* (95%*CI*)		HR (95%CI)	
All-cause mortality						
GNRI (Quartile)						
Q1(80–110)	Reference	<0.001	Reference	<0.0001	Reference	0.002
Q2(110–117.6)	0.58(0.44–0.78)		0.61(0.46–0.82)		0.60(0.44–0.84)	
Q3(117.6–127.6)	0.58(0.41–0.81)		0.59(0.42–0.83)		0.54(0.32–0.90)	
Q4(127.6–212.4)	0.51(0.38–0.69)		0.50(0.37–0.67)		0.40(0.22–0.72)	
Cardiovascular mortality						
GNRI (Quartile)						
Q1(80–110)	Reference	0.037	Reference	0.004	Reference	0.01
Q2(110–117.6)	0.56(0.36–0.87)		0.58(0.37–0.91)		0.59(0.37–0.93)	
Q3(117.6–127.6)	0.54(0.31–0.95)		0.48(0.26–0.88)		0.33(0.12–0.85)	
Q4(127.6–212.4)	0.55(0.34–0.89)		0.47(0.30–0.74)		0.29(0.11–0.78)	

In Model 1, confounders were adjusted for race, age only; Model 2 was adjusted based on race, age, education level, smoking, insurance, alcohol consumption, and PIR; Model 3 adjusted for race, age, education level, smoking, PIR, BMI, insurance, hypertension, alcohol consumption, diabetes, CVD, Scr, NEU.

### 3.3 Linear relationship between GNRI and mortality risk

**Figure 2:**
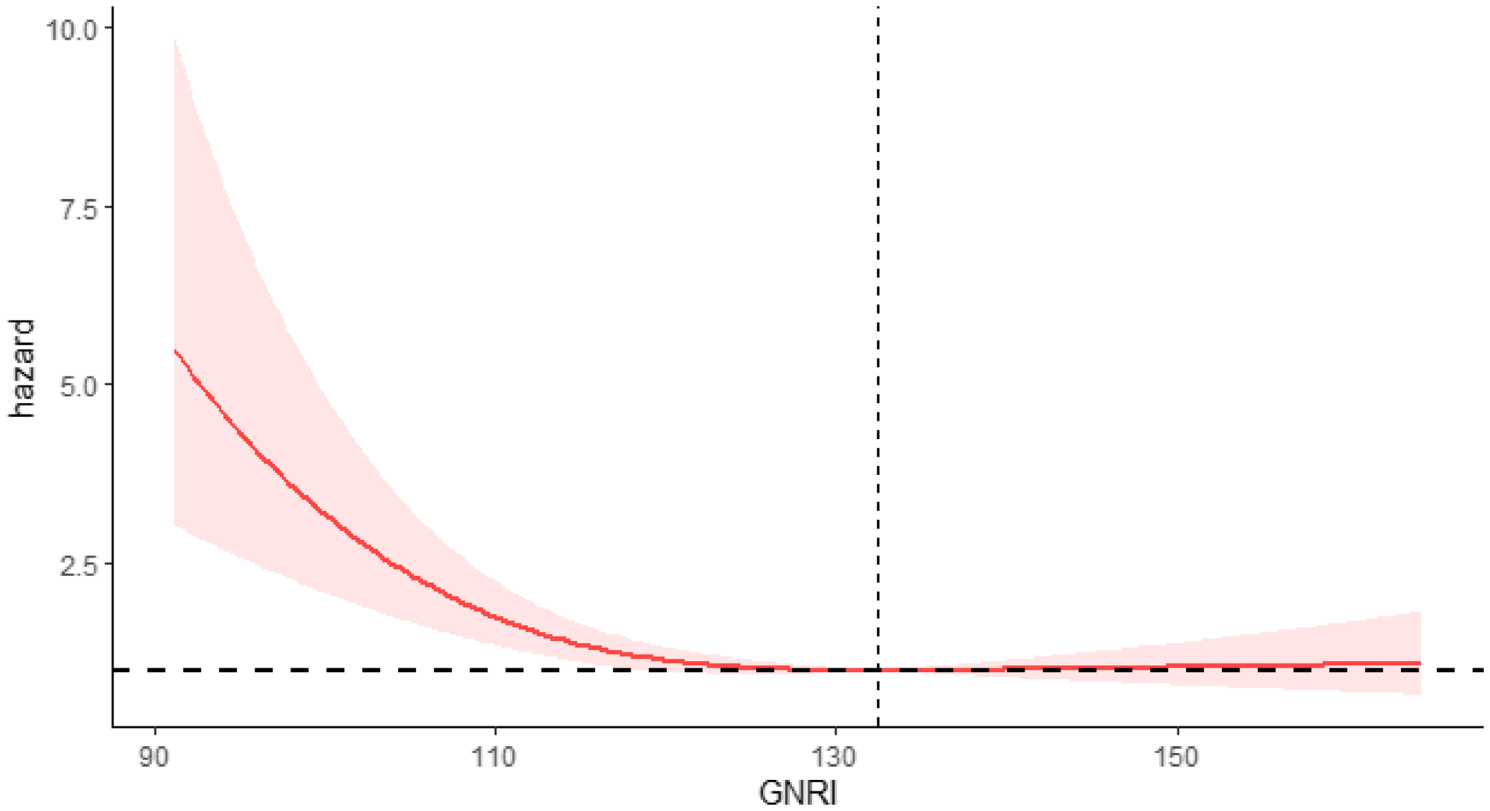
RCS showing the relationship between GNRI and all-cause mortality.

**Figure 3:**
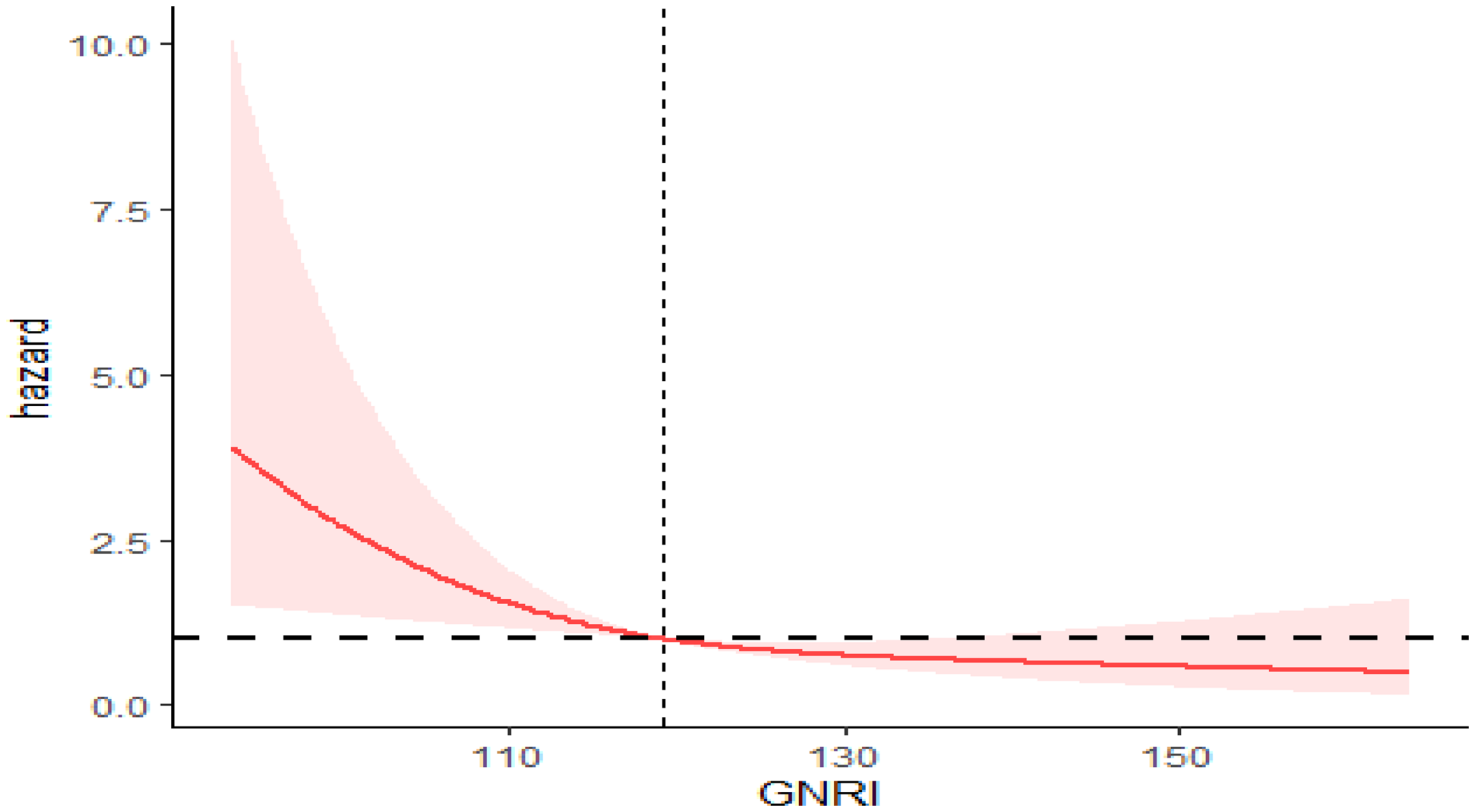
RCS showing the relationship between GNRI and cardiovascular mortality.

After controlling for covariates such as sex, race, marital status, age, education, smoking, PIR, BMI, insurance, hypertension, alcohol use, diabetes, and cardiovascular disease, restricted cubic spline (RCS) curves showed a non-linear negative correlation between GNRI and mortality risk. The relationship between GNRI and all-cause mortality is depicted by the RCS curve in Figure 2, which reveals a non-linear negative association between increasing GNRI and the risk of all-cause mortality (*P* for non-linearity <0.05). A similar non-linear negative relationship between GNRI and cardiovascular mortality risk is shown in Figure 3 (*P* for non-linearity <0.05).

### 3.4 Subgroup analysis

To investigate the relationship between GNRI and both all-cause and cardiovascular mortality, subgroup analyses were performed based on age, race, smoking status, alcohol use, and educational attainment. The results indicated no interaction effects between GNRI and the subgroups for either cardiovascular or all-cause mortality. Additionally, GNRI was not significantly associated with all-cause mortality in individuals who were former smokers or had a college education or higher (*p* > 0.05), nor was it significantly linked to cardiovascular mortality in individuals aged 60–70, non-drinkers, individuals of other races, former smokers, or current smokers (*p* > 0.05). These results suggest that, even after accounting for potential confounding factors, the relationship between higher GNRI quartiles and lower all-cause mortality risk remains consistent compared to the lowest GNRI quartile (Table 3).

**Table 3:** Subgroup analyses of GNRI and mortality.

Variable	All-cause mortality	*P value*	*P* for interaction	Cardiovascular mortality	*P value*	*p* for interaction
	*HR*(95%*CI*)			*HR* (95%*CI*)		
Age group			0.5			0.73
60–70	0.41(0.25–0.67)	0.001		0.52(0.26–1.04)	0.1	
≥70	0.41(0.27–0.61)	<0.0001		0.43(0.21–0.88)	0.03	
Drink			0.25			0.48
Yes	0.36(0.25–0.50)	<0.0001		0.32(0.17–0.62)	0.001	
No	0.41(0.21–0.82)	0.005		0.54(0.18–1.62)	0.37	
Race/ethnicity			0.95			0.31
White race	0.66(0.51–0.84)	<0.001		0.41(0.20–0.82)	0.01	
Other race	0.66(0.55–0.79)	<0.0001		0.41(0.21–0.82)	0.14	
Smoking status			0.09			0.64
Non-smoker	0.66(0.53–0.83)	<0.001		0.34(0.14–0.83)	0.01	
Former smoker	0.86(0.65–1.13)	0.28		0.40(0.19–0.87)	0.1	
Current smoker	0.56(0.44–0.72)	<0.0001		0.56(0.13–2.50)	0.28	
Edu			0.09			0.22
High school diploma	0.42(0.22–0.80)	<0.0001		0.22(0.07–0.69)	0.01	
More than high school	0.38(0.22–0.66)	0.05		0.38(0.15–0.98)	0.04	
Lower than high school	0.31(0.20–0.47)	<0.0001		0.54(0.28–1.03)	0.02	

## 4 Discussion

In this cohort study, we assessed the nutritional status of 1,417 elderly female CIAD patients using the Geriatric Nutritional Risk Index (GNRI). Through multiple Cox regression models adjusting for various potential confounders, we identified GNRI as a novel marker for predicting both all-cause and cardiovascular mortality in elderly women with CIAD. Based on restricted cubic spline regression models, we found a significant association between GNRI and the risk of both all-cause and cardiovascular mortality. Moreover, subgroup analysis revealed that the correlation between GNRI and mortality risk in elderly women with CIAD persisted across various subgroups based on potential risk factors. These findings suggest that effective nutritional management may serve as a promising strategy to improve the prognosis of elderly female CIAD patients.

CIAD can affect extra-pulmonary organs, and malnutrition is one of its severe extrapulmonary manifestations. Previous studies and guidelines have indicated that as the severity of the disease progresses, malnutrition also occurs, signaling a poorer prognosis (28, 29). Malnutrition refers to a deficiency of nutrients, such as calories and proteins, that negatively impact body structure and/or function. The onset of malnutrition in CIAD patients may be linked to increased energy expenditure from respiration, reduced physical activity, and depression-related appetite loss (30). Changes in inflammatory cytokines, hormonal levels, and adipokines also play a role in nutritional regulation in CIAD patients (31, 32). The negative nitrogen balance resulting from the imbalance between nutrient intake and energy consumption leads to a decline in muscle function, including that of skeletal muscles and the diaphragm. Simultaneously, inflammation and oxidative stress triggered by malnutrition play a central pathological role in the progression of CIAD, impairing immune defense and significantly increasing the risk of lung infections, which are major contributors to heightened mortality risk (33). In summary, poor nutritional status may have far-reaching effects on the risk of CIAD and overall health outcomes, necessitating timely nutritional management to prevent poor prognosis due to malnutrition. Studies on the effects of nutrients and antioxidants suggest that consuming foods rich in antioxidants and anti-inflammatory nutrients can effectively enhance antioxidant and anti-inflammatory capacities (34, 35), thereby promoting lung health and reducing CIAD risk (36, 37).

GNRI, developed by Bouillanne et al. (14) is a novel nutritional index that combines ideal body weight, current body weight, and serum albumin—three readily accessible indicators. It overcomes the limitations of using single indicators and more accurately reflects nutritional status (14). In a study evaluating elderly in patients with acute exacerbations of COPD (AECOPD), Zhang et al. (38) found that GNRI, as a quantitative nutritional tool, demonstrated high sensitivity (89.5%), satisfactory specificity (77.2%), and a large AUC value (0.834) for detecting nutritional risk in elderly AECOPD patients. A study by Chai X et al. investigated the relationship between GNRI and all-cause mortality in individuals aged 18 and above, revealing an association between malnutrition and higher all-cause mortality rates in COPD patients (39). In a retrospective cohort study, Wang et al. (40) found that GNRI was an independent predictor of 90-day mortality in elderly ICU patients with COPD, identifying a GNRI cutoff point of 101.5. Our findings align with previous studies, but our research focused on CIAD, encompassing not only COPD but also asthma and chronic bronchitis. With the increasing prevalence and mortality of CIAD among women, our study focused on elderly female patients, revealing a more targeted relationship between GNRI and mortality risk. We also found that these relationships were nonlinear, and subgroup analyses further confirmed the robustness of the results. A large cross-sectional study in the UK found that higher serum albumin levels were associated with a lower risk of COPD, with a risk ratio of 0.74 (95% CI: 0.67–0.81) (41). Serum albumin, a multipurpose plasma protein that makes up more than half of all plasma proteins, has significant antioxidant qualities. A major contributing factor to the development of CIAD is inflammation, which is exacerbated by oxidative stress (42). Higher albumin levels help enhance antioxidant capacity, protecting tissues from inflammation-related damage (43). It is well recognized that obesity is often linked to an increased incidence of chronic diseases. However, studies have indicated that a BMI below 18.5 is a risk factor for COPD (44, 45), while being overweight or obese (BMI ≥25 kg/m^2^) may reduce the incidence of COPD (45, 46). These combined factors may explain why GNRI has predictive value for disease-related mortality risks.

Furthermore, our study revealed a significant relationship between GNRI and the risk of CIAD and all-cause mortality in elderly women. Genetic studies on airway diseases have suggested a gender-related genetic component in the prevalence of airway diseases (47), with women being more vulnerable to smoking-related lung damage and experiencing more severe inflammatory responses. Epidemiological evidence indicates that female patients with chronic respiratory diseases demonstrate greater disease severity and are at significantly higher risk of acute exacerbations compared to their male counterparts (48, 49). Unfortunately, the specific mechanisms behind these phenomena remain unclear and require further investigation.

Our study has several strengths. First, we utilized a large, real-world sample from the NHANES database, which includes long-term follow-up data on elderly female CIAD patients, making it a prospective study. All patients were from the NHANES survey, minimizing selection bias and more closely reflecting real-world experiences. Second, we implemented three robust adjustment models in this prospective study, effectively reducing potential residual confounding factors. Nevertheless, the main limitations of this study include the possibility of unmeasured or unaccounted-for confounding factors, such as CIAD comorbidities or medication use, that may have influenced the observed outcomes. Additionally, the classification based on GNRI score distribution may reduce statistical reliability, but to address this limitation, we employed the RCS method to examine the linear relationship between GNRI and elderly female CIAD patients. Finally, our study focused solely on CIAD, without separately exploring the risks associated with COPD, asthma, and chronic bronchitis. Future research should refine these aspects. Lastly, as the study was conducted on a representative sample in the United States, this may limit the generalizability and applicability of our findings.

## 5 Conclusion

In conclusion, this study indicates that, in elderly female patients with CIAD, the risk of malnutrition, as assessed by the GNRI score, is negatively correlated with both cardiovascular and all-cause mortality. Based on these findings, GNRI may serve as a useful tool for predicting the prognosis of elderly female CIAD patients. Future cohort studies or randomized controlled trials are urgently needed to validate these results and provide more precise and effective strategies for disease prevention, management, and mortality reduction.

## Data Availability

The original contributions presented in the study are included in the article/supplementary material, further inquiries can be directed to the corresponding authors.

## Footnotes

^1^ https://wwwn.cdc.gov/nchs/nhanes/continuousnhanes/documents.aspx?BeginYear=2017&utm_source=chatgpt.com

^2^ https://www.cdc.gov/nchs/data-linkage/mortality-public.htm
